# Anti-MDA5 autoantibodies in juvenile dermatomyositis identify a distinct clinical phenotype: a prospective cohort study

**DOI:** 10.1186/ar4600

**Published:** 2014-07-02

**Authors:** Sarah L Tansley, Zoe E Betteridge, Harsha Gunawardena, Thomas S Jacques, Catherine M Owens, Clarissa Pilkington, Katie Arnold, Shireena Yasin, Elena Moraitis, Lucy R Wedderburn, Neil J McHugh

**Affiliations:** 1 Royal National Hospital for Rheumatic Diseases, Bath, UK; 2 Department of Pharmacy and Pharmacology, University of Bath, Bath, UK; 3 Department of Rheumatology, North Bristol NHS Trust, Bristol, UK; 4 Department of Histopathology, Great Ormond Street Hospital NHS Foundation Trust, London and Neural Development Unit, University College London, London, UK; 5 Department of Cardiothoracic Radiology, Great Ormond Street Hospital NHS Foundation Trust, London, UK; 6 Rheumatology Unit, Great Ormond Street Hospital NHS Foundation Trust, London, UK; 7 Rheumatology Unit UCL Institute of Child Health, London, UK; 8 Rheumatology Unit UCL Institute of Child Health and Arthritis Research UK Centre for Adolescent Rheumatology at UCL, UCLH and GOSH l, London, UK; 9 Royal National Hospital for Rheumatic Diseases, Bath, BA1 1RL, Bath, UK

## Abstract

**Introduction:**

The aim of this study was to define the frequency and associated clinical phenotype of anti-MDA5 autoantibodies in a large UK based, predominantly Caucasian, cohort of patients with juvenile dermatomyositis (JDM).

**Methods:**

Serum samples and clinical data were obtained from 285 patients with JDM recruited to the UK Juvenile Dermatomyositis Cohort and Biomarker Study. The presence of anti-MDA5 antibodies was determined by immunoprecipitation and confirmed by ELISA using recombinant MDA5 protein. Results were compared with matched clinical data, muscle biopsies (scored by an experienced paediatric neuropathologist) and chest imaging (reviewed by an experienced paediatric radiologist).

**Results:**

Anti-MDA5 antibodies were identified in 7.4% of JDM patients and were associated with a distinct clinical phenotype including skin ulceration (*P* = 0.03) oral ulceration (*P* = 0.01), arthritis (*P* <0.01) and milder muscle disease both clinically (as determined by Childhood Myositis Assessment Score (*P* = 0.03)) and histologically (as determined by a lower JDM muscle biopsy score (*P* <0.01)) than patients who did not have anti-MDA5 antibodies. A greater proportion of children with anti-MDA5 autoantibodies achieved disease inactivity at two years post-diagnosis according to PRINTO criteria (*P* = 0.02). A total of 4 out of 21 children with anti-MDA5 had interstitial lung disease; none had rapidly progressive interstitial lung disease.

**Conclusions:**

Anti-MDA5 antibodies can be identified in a small but significant proportion of patients with JDM and identify a distinctive clinical sub-group. Screening for anti-MDA5 autoantibodies at diagnosis would be useful to guide further investigation for lung disease, inform on prognosis and potentially confirm the diagnosis, as subtle biopsy changes could otherwise be missed.

## Introduction

Autoantibodies can be detected in approximately 60% of children with juvenile dermatomyositis (JDM) [1]. Anti-MDA5 was initially identified in adult Japanese patients with clinically amyopathic myositis and interstitial lung disease (ILD), a phenotype more commonly seen in East-Asia [1,2]. Studies based in East-Asia have identified anti-MDA5 (anti-Melanoma differentiation associated gene 5) autoantibodies in 19 to 35% of adult dermatomyositis (DM) patients [2,3], where it is associated with clinically amyopathic myositis (81%), rapidly progressive ILD (74%) and a poor prognosis [4,5]. Anti-MDA5 has been identified at a lower frequency in adult Caucasians [6-8], where patients appear to have little myositis and an increased risk of ILD, but not rapidly progressive ILD. Unlike in East-Asian patients, in predominantly Caucasian adult populations a characteristic cutaneous phenotype has been described, which includes skin ulceration and painful palmar papules [2,6,8]. Patients can present with inflammatory arthritis, and similarities to the anti-synthetase syndrome have been described [8].

The phenotype of children with anti-MDA5 has yet to be clearly established. A small Japanese study identified anti-MDA5 in 38% of JDM patients, all of whom had ILD, and three patients with very high titres of anti-MDA5 had rapidly progressive ILD [9]. A further study of 35 Japanese JDM patients demonstrated a significant association between the presence of anti-MDA5 and the development of rapidly progressive ILD [10]. Although data on pulmonary involvement in JDM are limited, reported rates of ILD in UK and USA patients with JDM are far lower than in adult DM [11]. The incidence in Japanese JDM populations, however, has been reported to approach 50% [12]. This may reflect genetic and environmental differences or differences in autoantibody prevalence (and therefore associated complications) between these populations. Here we report the clinical and muscle histology associations of anti-MDA5 autoantibodies in a large cohort of predominantly Caucasian patients with juvenile-onset myositis.

## Methods

### Patients

Patient serum samples and clinical data were obtained through the UK Juvenile Dermatomyositis Cohort and Biomarker Study (JDCBS). The JDCBS is a large cohort of UK patients with myositis, the majority with JDM [13]. Patients are recruited consecutively on presentation to paediatric rheumatology departments across the UK, and data are collected prospectively. Children and young people aged 16 years or under are included based on a diagnosis of definite or probable JDM or polymyositis by Bohan and Peter criteria [14], as well as JDM, or polymyositis with overlapping connective-tissue disease features. Biological specimens, including sera and muscle biopsies, are collected and stored as described [13]. Ethical approval has been obtained from the North Yorkshire Multicentre Research ethics committee and this study was also approved by the steering committee of the JDCBS. Parental consent for children, and consent or age-appropriate assent was obtained for all patients in accordance with the declaration of Helsinki. Clinical data were collected on standardised proformas: data items in this analysis included demographics, the presence of ulceration, oedema, calcinosis and/or arthritis during the follow-up period, in addition to JDM disease activity measures (childhood myositis assessment score (CMAS), manual muscle testing score (MMT), serum creatinine kinase, presence of skin disease and physician global visual analogue score (PGAS). When available, muscle biopsy samples and radiographic or computed tomography (CT) images were analysed.

Remission in JDM was defined as a full-strength CMAS >48 [15], the absence of skin disease (no rash, Gottron’s papules, oedema, or ulceration) and a PGAS <1. Whilst this definition of remission has not been validated all features listed are standard outcome measures in JDM. Where possible we also utilised the recently proposed Paediatric Rheumatology International Trials Organisation (PRINTO) criteria for disease inactivity in JDM, defined as at least three out of the four following criteria; creatinine kinase ≤150, CMAS ≥48, MMT ≥78 and PGAS ≤0.2 [16].

#### MSA detection

Serum or plasma was available from 285 patients. Samples were all stored at −80°C prior to analysis. Immunoprecipitation of radiolabelled K562 cells was performed to determine the presence of autoantibodies. The presence of anti-MDA5 was confirmed using ELISA.

#### Immunoprecipitation

Sera (10 μl) was mixed with 2 mg protein-A-Sepharose beads (Sigma, UK) in IPP buffer (10 mM Tris-Cl pH 8.0, 500 mM NaCl, 0.1% v/v Igepal) at room temperature for 30 minutes. Beads were washed in IPP buffer prior to the addition of 120 μl (^35^S)methionine-labelled K562 cell extract in IPP buffer. Samples were mixed at 4°C for 2 h. Beads were washed in IPP buffer and TBS (10 mM Tris-Cl pH 7.4, 150 mM NaCl) before being resuspended in 50 μl SDS sample buffer (Sigma, UK). After heating, proteins were fractionated by 9% SDS-PAGE gels, enhanced, fixed and dried. Labelled proteins were analysed by autoradiography.

#### ELISA

ELISAs were performed as previously described [2] with some modifications: 96-well polystyrene plates were coated with rMDA-5 (Origene, USA) at 4°C for 16 h. Serum samples were diluted to 1:250. Secondary antibodies were conjugated goat anti-human IgG/M antibodies (Sigma, UK). Tetramethybenzidine liquid substrate (Sigma, UK) was then added (10-minute incubation). All samples were tested in duplicate and optical density was read at 450 nm using an automatic plate reader. The negative cut off was defined as >3 SD above the mean in serum from 34 normal healthy (adult) controls.

#### Indirect immunofluorescence

Indirect immunofluorescence was performed on HEp-2 cells (Immunoconcepts, USA) according to manufacturers’ instructions. Samples were diluted to 1 in 40.

#### Immunoblot

Where ANA was positive, ENA (including anti-Ro52) were looked for by immunoblot according to manufacturer’s instructions, (ANA profile 5, Euroimmun, Germany).

#### Muscle Biopsy

Biopsies were processed, stained and scored using the International JDM score tool as described [17]. All biopsy scores were agreed by an experienced paediatric neuropathologist (TSJ) who was blinded to autoantibody and clinical data.

### Pulmonary involvement

Where available, chest imaging obtained during routine clinical care was reviewed by a single experienced thoracic radiologist (CO). Images were initially reviewed blind, following which, clinical data were made available, including pulmonary function tests (PFTs) and aspiration risk, to assist with interpretation.

### Statistical analysis

Statistical analysis was performed in SPSS. Potential differences between two groups were assessed using Chi-squared analysis with Yate’s continuity correction or Fisher’s exact test for groups with small numbers. The Mann-Whitney *U*-test was used to compare non-normally distributed continuous data.

## Results

Demographic data for the overall cohort and those with anti-MDA5 are shown in Table 1. Anti-MDA5 autoantibodies were identified in 7.4% of patients (21/285) and did not co-exist with other myositis-specific or associated autoantibodies. ANA was negative in 17 out of the 21 patients with anti-MDA5. The remaining four patients had a non-specific, fine-speckle, nucleolar-sparing ANA pattern. This pattern can be consistent with the presence of anti-Ro52, although anti-Ro52 was not present on immunoblot.

**Table 1:** Demographic characteristics of the 285 patients in this study

	**All JDM patients, n = 285**	**Anti-MDA5-positive patients, n = 21**
**Female, number (%)**	206 (72)	15 (71)
**Caucasian, number (%)**	220 (78)	16 (76)^b^
**Diagnosis, number (%)**^**a**^		
**Dermatomyositis**	242 (85)	21 (100)
**Polymyositis**	1 (0.4)	0
**Overlap**	33 (12)	0
**Age at disease onset, years, median (IQR)**	6.3 (IQR 4 to 10)	6.6 (IQR 4 to 10)
**Length of follow up, years, median (IQR)**	9 (IQR 5 to 12)	8 (IQR 5 to 11)
**Highest ever CK, u/l, median (IQR)**	220 (IQR 111 to 1132)	129 (88 to 157)

^a^Nine patients were classified as having focal myositis or other idiopathic inflammatory myopathy; ^b^One Black-African patient, one Indian patient, one Pakastani patient and two patients from other ethnic groups. All anti-MDA5 autoantibody-positive patients had juvenile dermatomyositis (JDM). Otherwise, demographic data did not differ significantly between anti-MDA5 antibody positive patients and the overall cohort. IQR, interquartile range; CK, creatine kinase.

### Cutaneous and joint disease

Anti-MDA5 autoantibodies were significantly associated with the occurrence of both skin ulceration (52% anti-MDA5 positive versus 27% anti-MDA5 negative, *P* = 0.03) and oral ulceration (71% anti-MDA5 positive versus 45% anti-MDA5 negative, *P* = 0.01) occurring at any time point during the follow-up period but not with oedema or calcinosis. As expected the large majority of all the JDM cases (76%) had Gottron’s papules and/or heliotrope rash (85%), but there were no significant associations between presence of anti-MDA5, and Gottron’s papules or heliotrope rash. Despite the association with skin ulcerations we noted that no child with anti-MDA5 autoantibodies had a history of bowel vasculitis. There were more children with arthritis in the anti-MDA5-positive group: 86% of those with anti-MDA5 autoantibodies had arthritis compared to 51% of those without (*P* <0.01). In those with anti-MDA5 and arthritis 46% had symmetrical polyarthritis involving the small joints of the hands.

### Muscle disease

The CMAS was used to assess muscle strength. Overall the lowest recorded CMAS was significantly higher (*P* = 0.03) in those with anti-MDA5 compared to the rest of the cohort, indicating less severe weakness (Table 2). A CMAS of 48 or above has previously been demonstrated to correspond to no significant functional weakness [15]. The presence of normal muscle enzymes (CK and lactate dehydrogenase (LDH)) and a lowest recorded CMAS ≥48 was significantly associated with anti-MDA5 antibodies (12.5% anti-MDA5-positive versus 1.2% anti-MDA5-negative, *P* = 0.05).

**Table 2:** Muscle disease in those with anti-MDA5

		**Anti-MDA5 autoantibody-positive**	**Anti-MDA5 autoantibody-negative**
**Muscle strength**			
Median lowest ever recorded CMAS (IQR)^a^		46 (38 to 52)	40 (27 to 48)
**Muscle histology**			
Median biopsy score (IQR) (17)^b^	Inflammatory (0 to 12)^c^	2 (2 to 4.8)	7 (5.5 to 9.5)
	Vascular (0 to 3)^c^	0 (0 to 0)	1 (0 to 2)
	Muscle fibre (0 to 10)^c^	2 (1, 2)	7 (4 to 9)
	Connective tissue (0 to 2)^c^	0 (0 to 0)	1 (0 to 1)
	**Total (0 to 27)**^**c**^	**4 (3.25 to 8.5)**	**15 (12.5 to 21)**
	**VAS severity (0 to 10)**^**c**^	**2** (2)	**5 (3.6 to 8)**

^a.^*P* = 0.03; ^b^biopsies were analysed from 11 patients with anti-MDA5 autoantibodies and 30 without; ^c^*P* <0.005. Patients with anti-MDA5 had less muscle involvement, both clinically, as measured by the childhood myositis assessment score (CMAS) and histologically, as quantified by the juvenile dermatomyositis (JDM) muscle biopsy scoring tool. VAS, visual analogue scale.

Muscle biopsies were available for 11 patients with anti-MDA5. These 11 biopsies, plus 30 others (randomly selected from JDM patients without anti-MDA5 autoantibodies) were scored using the previously published and validated JDM biopsy score tool, (Table 2) [17,18]. This tool assesses severity of pathological change in four domains (inflammatory, muscle fibre, vascular, connective tissue), leading to an overall score, and a score of 0 to 10.0 on a visual analogue scale (VAS) for assessment of severity [17,18].

Mean total biopsy scores and VAS scores for severity were significantly lower in those with anti-MDA5, (both *P* <0.001). The difference in total biopsy scores between the two groups lay in a more destructive histological pattern in the non-anti-MDA5 group with significant differences in score within all four domains (inflammatory, *P* = 0.001; vascular, *P* = 0.004; muscle fibre, *P* <0.001 and connective tissue, *P* <0.003).

### Pulmonary disease

Chest imaging had been performed in 12 patients with anti-MDA5 and 9 had imaging studies available for review (7 patients with CT and 2 with radiographs). Three patients had chest radiographs previously reported as showing no abnormality; these were not available to re-review. As chest imaging was performed as part of routine care it was variably timed post diagnosis (up to 68 months). A request for imaging generally coincided with either time of diagnosis or when the patient reported respiratory symptoms. No patients with abnormal PFTs went on to have high-resolution computed tomography (HRCT).

Two patients, both aged 8 years at diagnosis, had definite radiological changes consistent with ILD (as demonstrated on HRCT performed at 16 and 27 months post diagnosis). Both patients had abnormal PFTs, although in the later test, forced expiratory volume at 1 s (FEV1) and forced vital capacity (FVC) were only slightly reduced (78 and 86% predicted respectively) despite extensive changes on HRCT. DLCO was not performed. Two further patients aged 4 and 2 years at diagnosis had abnormal imaging probably consistent with ILD; one with ground-glass changes on chest radiography but with no further imaging or PFTs performed, and one with extensive reticular changes on CT with radiologic appearances consistent with ILD, aspiration and/or infection. For the latter patient, taken in the clinical context this was felt most likely to represent ILD. The incidence of ILD in this group, therefore, appears to lie between 10 and 19%, although this may be an underestimate as nine patients had no chest imaging available, and for some patients the available imaging was performed many months post diagnosis.

Where ILD was demonstrated the radiological appearance was consistent with non-specific interstitial pneumonia and some patients had elements of organising pneumonia. Histology was not available to confirm the disease pattern. Of the two patients with definite ILD on imaging, both had follow-up images available, which demonstrated significant radiological improvement following treatment with intravenous cyclophosphamide.

### Disease outcome

Disease outcome was assessed at 2 years (range 20 to 28 months) post diagnosis and again at the last clinic visit, where this occurred 4 or more years post diagnosis, (mean 7.1 years in the anti-MDA5-positive group and 7.9 years in the anti-MDA5-negative group). Data were not yet available at 2 years post diagnosis when children had been diagnosed with JDM less than two years previously, had been recruited into the study more than 20 years post diagnosis or had not been reviewed between 20 and 28 months post diagnosis. Information was available for 151 of 285 (53%) children at 2 years post diagnosis (12 with anti-MDA5) and 136 children (48%) at more than 4 years post diagnosis (9 with anti-MDA5 autoantibodies).

Using a modified definition of remission, (full strength CMAS of >48 [15], the absence of skin disease and a PGAS <1), more patients with anti-MDA5 were in remission 2 years post-diagnosis (*P* = 0.04) than those without MDA5 autoantibodies, and there was a trend for more of those with anti-MDA5 to be off all medication at 2 years post diagnosis (*P* = 0.07). We also analysed disease activity using the recently proposed PRINTO definition of disease inactivity in JDM [16]. Despite smaller numbers with complete data for this analysis, the results were concordant, with more children with anti-MDA5 autoantibodies in remission at 2 years post diagnosis (*P* = 0.02), Table 3.

**Table 3:** Outcome at 2 and >4 years post diagnosis for affected children with and without anti-MDA5 antibodies

	**Two years post diagnosis**		**More than four years post diagnosis**^**a**^	
	**Anti-MDA5 present**	**Anti-MDA5 absent**	**Anti-MDA5 present**	**Anti-MDA5 absent**
Inactive disease	6 (50%)^b^	30 (22%)	6 (66.6%)	59 (46%)
Active disease	6 (50%)^b^	109 (78%)	3 (33.3%)	68 (54%)
PRINTO inactive disease	7 (87%)^b^	44 (43%)	4 (100%)	51 (71%)
PRINTO active disease	1 (13%)^b^	59 (57%)	0 (0%)	21 (29%)

Results are presented as number of patients (%). ^a^Average 7.1 years anti-MDA5-positive and 7.9 years in anti-MDA5-negative; ^b^*P* <0.05. Inactive disease is defined as childhood myositis assessment score (CMAS) >48, absent skin disease and physician global assessment score (PGAS) <1. Paediatric Rheumatology International Trials Organisation (PRINTO) criteria for inactivity is defined as at least three of the following; creatinine kinase (CK) ≤150, CMAS ≥48, manual muscle testing score ≥78 and PGAS ≤0.2. Patients with anti-MDA5 were more likely to have inactive disease at the time points analysed. This was statistically significant at 2 years post diagnosis.

Given the association of anti-MDA5 autoantibodies with ulceration and ILD, which are both considered to be features of severe disease, we investigated whether these patients were more likely to be targeted for aggressive treatment. Slightly more patients with anti-MDA5 had received cyclophosphamide treatment than those without but this difference was not significant, with 29% compared to 21% of those without. Likewise no significant difference was seen in the proportion receiving methotrexate treatment (90% of those with anti-MDA5 versus 93% without). At 4 or more years post diagnosis no statistically significant difference in disease activity was seen but more children with anti-MDA5 had inactive disease (Table 3).

## Discussion

The identified prevalence of anti-MDA5 in 7.4% of the patients should be considered a minimum, as whilst the earliest available serum sample was used for autoantibody detection, this was often months post diagnosis. Sato *et al*. demonstrated a fall in anti-MDA5 titre in response to treatment and it is therefore possible that some autoantibody-negative patients in our cohort were treated patients with anti-MDA5 [19].

Anti-MDA5 autoantibodies were first described in adults with amyopathic myositis and rapidly progressive ILD: amyopathic myositis is recognised in children but it is rare, and more often patients have mild or progressive muscle disease [20,21]. We have previously shown that histological features on biopsy are very mild in cases of amyopathic JDM, and include absence of upregulation of major histocompatibility complex (MHC) class I protein, which is usually found in JDM biopsy tissue from more typical cases [22]. The data presented here suggest that in JDM anti-MDA5 antibodies are associated with mild muscle involvement, both clinically and histologically. To our knowledge this is the first report of quantitative biopsy scoring in an autoantibody-specific subgroup and suggests that in addition to clinical phenotype, autoantibodies may also reflect variations in underlying muscle pathology.

Similar to previous studies in US and European adults, anti-MDA5 autoantibodies are associated with less severe muscle disease and a characteristic cutaneous phenotype, and in particular, ulcerative skin disease [6,23]. Whilst skin ulceration is generally considered one of the most severe cutaneous manifestations of JDM, there was no significant association with other severe cutaneous disease features, such as oedema or calcinosis. The association with arthritis and anti-MDA-5 antibodies has also been described in US adults [6,8]: in one study several adult patients had even initially been suspected to have rheumatoid arthritis [8]. Although we did not analyse disease presentation in this study, it seems likely that some juvenile patients could be similarly misclassified. We suspect that this sub-group of patients may often present as a diagnostic conundrum with rash, ulceration and polyarthritis but minimal muscle disease.

Anti-MDA5 autoantibodies have been associated with a poor prognosis in adult East-Asian cohorts due to the association with rapidly progressive ILD [4,24]. This has also been described in Japanese juvenile-onset patients with anti-MDA5 [9]. In our cohort, 19% of those with anti-MDA5 had radiological evidence of ILD. A large US study of clinical phenotype of juvenile myositis patients reported an incidence of ILD of 4.8% in those with JDM [25]. Anti-MDA5 antibodies in our cohort were mutually exclusive and specifically no patient had additional anti-synthetase antibodies. It thus appears highly probable that anti-MDA5 is associated with ILD in JDM: larger future studies will be required to confirm this.

One limitation of this study is that not all of the cases in this study had chest imaging available to review and where imaging was available this was often some months post diagnosis and following treatment. Furthermore, radiograhy is an insensitive tool in excluding ILD, and approximately 10% of patients with ILD detected on HRCT have a normal chest radiograph, especially early in the disease [26]. While our estimates of the prevalence of ILD in the anti-MDA5 subpopulation may therefore be an underestimate, particularly if some children have mild ILD, which responds to standard therapies, it does accurately represent the proportion with ILD diagnosed during routine clinical practice. A retrospective study of pulmonary outcome in JDM in a Norwegian population reported a previous diagnosis of ILD in 3 out of 59 patients (5%), one of whom was known to have anti-Jo1 antibodies [27]. A further four patients, however, were identified as having evidence of ILD on subsequent HRCT. Anti-MDA5 antibody status was not reported but interestingly these four patients all had arthritis and skin ulcers [27]. Anti-synthetase autoantibodies, which are associated with ILD, are rare in JDM. Interestingly, of the six patients in our cohort with anti-synthetase autoantibodies, four had evidence of ILD, suggesting that they confer a greater risk of developing ILD than anti-MDA5, although the incidence in those with anti-Jo-1 autoantibodies (three patients) was similar (33%).

Our findings also highlight the relative infrequency of chest imaging by CT in children with JDM, in the UK, in part secondary to concerns with regard to radiation exposure and the perceived low incidence of lung disease. Assessment for the presence of anti-MDA5 autoantibodies would be useful to help target patients in whom careful repeat chest imaging may be recommended. This would be particularly valuable in patients diagnosed at a young age, when PFTs cannot be performed.

Importantly and similar to adult Caucasian patients with anti-MDA5 [6,8], no patients had rapidly progressive ILD. Furthermore, the response to treatment appeared to be good with significant improvement seen on follow-up imaging where this was available.

Interestingly, despite associations with ulceration and probably ILD, both considered poor prognostic features, significantly more children with anti-MDA5 were in remission at 2 years post diagnosis than the rest of the cohort.

At present diagnostic testing for anti-MDA5 is available at a limited number of centres worldwide. Standard laboratory techniques, including indirect immunofluorescence, are of limited benefit and are likely to provide negative or non-specific results. Whilst one study did report a granular/reticular cytoplasmic speckle in anti-MDA5 positive patients [28] this has not been our experience and may depend on the exact technique used. Furthermore, this pattern is non-specific and may not be reported by many laboratories. An association between anti-Ro52 and anti-MDA5 has been noted in two adult studies [6,8]. We did not identify co-existent anti-Ro52 autoantibodies in any of our patients with anti-MDA5. There is growing interest in more widespread testing for myositis-specific autoantibodies using standard laboratory techniques: line blot kits, which include anti-MDA5, have been developed and will shortly be commercially available. We anticipate that in the future testing for the presence of anti-MDA5 autoantibodies where JDM or JDM overlap-syndromes are suspected will become standard clinical practice.

## Conclusion

Anti-MDA5 autoantibodies can be identified in a small but significant proportion of UK patients with JDM. The particular clinical phenotype of this subgroup is similar to that described in predominantly Causcasian adult cohorts but appears to differ from that described in East-Asian populations. The identification of anti-MDA5 at diagnosis would be useful to guide further investigation for possible lung disease, inform on prognosis and potentially to confirm the diagnosis, as subtle biopsy changes could otherwise be missed.

## Abbreviations

ANA: antinuclear antibody; CMAS: childhood myositis assessment score; CT: computed tomography; DLCO: diffusion capacity; ELISA: enzyme-linked immunosorbent assay; ENA: extractable nuclear antigen; HRCT: high-resolution computed tomography; ILD: interstitial lung disease; JDCBS: Juvenile Dermatomyositis Cohort and Biomarker Study; JDM: juvenile dermatomyositis; MMT: manual muscle testing score; PFT: pulmonary function test; PGAS: physician global assessment score; PRINTO: Paediatric Rheumatology International Trials Organisation; VAS: visual analogue scale.

## Competing interests

The authors declare no conflicts of interest.

## Authors’ contributions

ST serotyped samples, analysed the data, and drafted and prepared the manuscript. ZB serotyped samples and optimised the ELISA technique. HG serotyped samples. TJ and SY reviewed and scored muscle biopsies. CO reviewed and reported chest imaging. KA extracted clinical data. EM reviewed chest imaging and extracted clinical data. TJ, CO, CP, LW and NM reviewed and revised the manuscript, provided academic input assisted with data interpretation. All authors read and revised the final manuscript.
